# Induced Pluripotent Stem Cells as Vasculature Forming Entities

**DOI:** 10.3390/jcm8111782

**Published:** 2019-10-25

**Authors:** Antonio Palladino, Isabella Mavaro, Carmela Pizzoleo, Elena De Felice, Carla Lucini, Paolo de Girolamo, Paolo A. Netti, Chiara Attanasio

**Affiliations:** 1 CESMA—Centro Servizi Metrologici e Tecnologici Avanzati, University of Naples Federico II, 80146 Naples, Italy; 2 Department of Veterinary Medicine and Animal Productions, University of Naples Federico II, I-80137 Naples, Italy; 3 Interdepartmental Center for Research in Biomaterials (CRIB) University of Naples Federico II, I-80125 Naples, Italy; 4 School of Biosciences and Veterinary Medicine, University of Camerino, 62032 Camerino, MC, Italy; 5 Center for Advanced Biomaterials for Healthcare, Istituto Italiano di Tecnologia, 80125 Naples, Italy

**Keywords:** induced pluripotent stem cells, tissue engineering, angiogenesis, tissue regeneration, from bench to bedside

## Abstract

Tissue engineering (TE) pursues the ambitious goal to heal damaged tissues. One of the most successful TE approaches relies on the use of scaffolds specifically designed and fabricated to promote tissue growth. During regeneration the guidance of biological events may be essential to sustain vasculature neoformation inside the engineered scaffold. In this context, one of the most effective strategies includes the incorporation of vasculature forming cells, namely endothelial cells (EC), into engineered constructs. However, the most common EC sources currently available, intended as primary cells, are affected by several limitations that make them inappropriate to personalized medicine. Human induced Pluripotent Stem Cells (hiPSC), since the time of their discovery, represent an unprecedented opportunity for regenerative medicine applications. Unfortunately, human induced Pluripotent Stem Cells-Endothelial Cells (hiPSC-ECs) still display significant safety issues. In this work, we reviewed the most effective protocols to induce pluripotency, to generate cells displaying the endothelial phenotype and to perform an efficient and safe cell selection. We also provide noteworthy examples of both in vitro and in vivo applications of hiPSC-ECs in order to highlight their ability to form functional blood vessels. In conclusion, we propose hiPSC-ECs as the preferred source of endothelial cells currently available in the field of personalized regenerative medicine.

## 1. Introduction

The main goal of tissue engineering (TE) is to replace tissues and, more ambitiously, organs damaged by a large variety of insults. To this aim, TE relies on the combination of biocompatible scaffolds, suitable cellular sources and correct sets of signaling molecules. The integration of these factors is required for a successful and long-lasting regeneration process. The field is continuously evolving and the number of both in vitro and in vivo studies has grown exponentially over the last two decades. Despite this substantial increase still a very small fraction of bioengineered products is currently used for clinical applications.

The reason behind this discrepancy is mainly related to factors that cause graft failure, thus influencing the clinical translatability. It has been widely demonstrated that graft failure is mostly caused by the inadequate onset of a functional vasculature within the implanted scaffold. The insufficient vascularization of the neoforming tissue leads to a lack of integration of the construct with the host tissue due to insufficient metabolic supply and waste disposal [1]. In this scenario, different strategies have been developed, relying on the use of bioactive molecules [2], specific architectures [3] and topographic signals [4,5]. Concerning the support to vascular growth with bioactive factors [6] it has been proven that, in some cases, the host vasculature itself is unable to extend into the core of scaffolds exceeding 200 µm in thickness [7]. 

**Figure 1:**
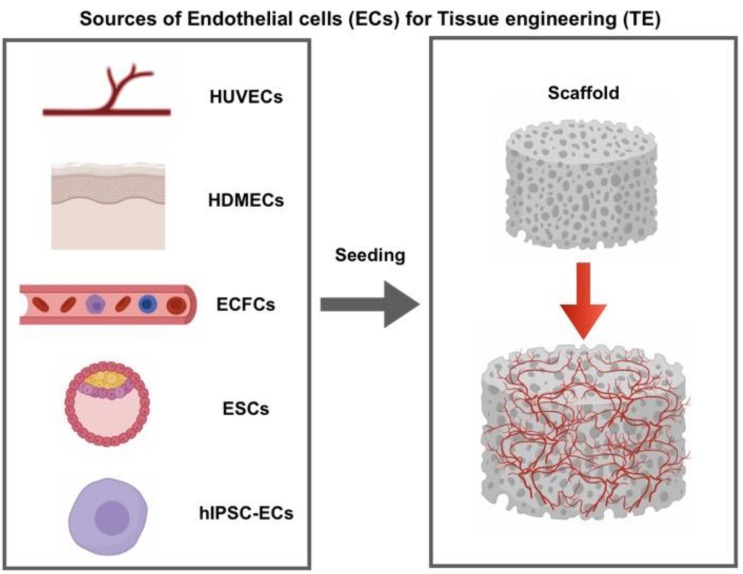
Sources of endothelial cells (ECs) used in scaffold-based approaches for tissue engineering (TE). HUVECs: Human Umbilical Vein Endothelial Cells, HDMECs: Human Dermal Microvascular Endothelial Cells, ECFCs: Endothelial Colony Forming Cells, ESCs: Embryonic Stem Cells, hIPSC-ECs: Endothelial Cells derived from Human Induced Pluripotent Stem Cells.

A possible approach to overcome this drawback is based on the incorporation of vasculature forming cells, namely endothelial cells (Figure 1), into the scaffold, as it has been already successfully performed in the case of bioengineered tissues [8,9] and organs [10]. ECs for scaffold vascularization could be derived from multiple sources. Doubtless, in most studies, the cells used are human umbilical vein endothelial cells (HUVECs), which hold several features that make them an attractive source of primary human ECs. They are retrieved from the umbilical cord, a tissue which is usually discarded, and is thus relatively abundant and easy to isolate [11]. In addition, a large set of assays has been set-up and widely validated. This means that a broad range of standardized tools to study angiogenic and antiangiogenic factors is available. Furthermore, a developing understanding of the cascade of molecular and cellular mechanisms of angiogenesis is crucial [12]. On the other hand, HUVECs show high heterogeneity depending on the donor, beyond the rapid loss of endothelial phenotype that they show when they are kept in culture [13]. The latter issue is extremely limiting in the view of an autologous cell transplant. Therefore, alternative EC sources are urgently needed for tissue engineering applications.

In addition, adult tissues such as skin, adipose tissue and aorta or coronary arteries could also provide ECs [14]. From the beginning of the 2000s, several studies using mouse models have indicated that microvascular endothelial cells isolated from human dermal tissue (HDMECs) are able to generate a functional vascular network anastomosed with the host vasculature [15,16]. In the following years, several works using scaffold entrapped growth factors in combination with HDMECs further confirmed the ability of these cells to form a fully functional vascular network [17]. Thus, ECs derived from adult tissues represent a good alternative to HUVECs. However, these cells suffer some major limitations that impair their translatability into the clinic. In particular, tissue procurement requires a procedure that is invasive for the patient; in addition, the in vitro proliferative potential of the isolated cells is very low. These limitations demonstrate the necessity to find an alternative source of cells suitable to be used in regenerative medicine.

Advances in vascular biology shed light on putative HUVEC substitutes: Asahara et al. showed the presence of Endothelial progenitor cells in 1997 [18], and a few years later, in 2000, Lin et al. identified these cells in peripheral blood, indicating them as Endothelial colony forming cells (ECFCs) [19]. ECFCs show a full set of endothelial cell markers. Beyond the molecular similarity to adult endothelial cells, ECFCs also hold a functional competence specific to ECs. In fact, in vitro studies demonstrate that ECFCs are capable to form more efficient vascular networks when embedded in a collagen matrix in comparison to other EC sources [20]. Within the in vivo setting, ECFCs display the ability to integrate and form perfused blood vessels when injected into immunocompromised mice [21,22]. Furthermore, Fuchs reported that these cells are able to guide the vascularization of an engineered bone tissue equivalent [23]. Although, starting from their first identification, the use of ECFCs has constantly increased [24], this EC source also implies severe restrictions. Indeed, if on one hand ECFCs are an efficient source of autologous ECs, on the other their use is strongly limited by their amount in the peripheral blood, where only 0.05–0.2 cells/Ml can be retrieved [25]. In addition, Mund et al. indicated the absence of specific markers, which is a further significant hindrance to the widespread use of these cells [26]. Overall, these concerns strongly discourage the isolation of ECFCs for tissue engineering purposes [26]. In this context, the best source of ECs is probably represented by embryonic stem cells (ESCs) derived from the Inner Cell Mass (ICM) of the blastocysts. ESCs are able to remain undifferentiated and to indefinitely proliferate in vitro, while maintaining the potential to differentiate into derivatives of all three embryonic germ layers [27]. The use of human ESCs is strongly hampered by ethical concerns since the withdrawal of ICM results in the disruption of a human embryo. In this respect, even though several in vivo studies demonstrate their validity in forming new vessels, ESCs do not represent the ideal source of endothelial cells suitable for biomedical applications [28,29]. A remarkable breakthrough in cellular biology research was the discovery of induced pluripotent stem cells (iPSC) made by Takahashi in 2006 [30]. In adult life, multipotent stem cells can differentiate and replace almost all damaged tissues. Multipotency confers the ability to differentiate into cell lines belonging to the same germ layer. Pluripotent cells show a wider differentiation range; the germ layer of origin makes them even more exploitable for TE purposes. Yamanaka et al. [30] set up a method to reprogram mouse fibroblasts into iPSC by retroviral delivery of four reprogramming factors (OSKM factors: OCT-3/4, Sox2, Klf4 and c-Myc) while Takahashi et al., in 2007, improved the reprogramming method in order to obtain iPSC from human somatic cells (hiPSCs) [31]. The advent of iPSC represented a real milestone in the field of vascular biology since 2009, when Taura et al. collected the first evidence of the possibility to generate endothelial cells starting from iPSC (iPSC-EC) [32]. IPSC-ECs could be a valuable source of cells in regenerative medicine for several reasons [33]. These cells display the same pluripotency of ESCs, based on their gene expression profile, overcoming all the limitations that hampered embryonic stem cell usage [34,35]. Further iPSCs can be easily generated from patients; therefore, they can provide an autologous source of cells for regenerative medicine applications able to bypass the issue of host immune rejection. Moreover, iPSCs, as they are derived from adult somatic cells, do not present strong ethical concerns as ESCs do.

Among the various advantages, the most promising one is to have a tissue specific EC source; in fact, iPSC-EC display the same plasticity of immature ECs [36]. Evidence collected in independent studies demonstrates that, when exposed to tissue-specific cues, iPSC-ECs generate mature ECs able to almost completely resemble the characteristics of resident ECs [37,38]. On the other hand, a well-defined selection of iPSC is required, since, once implanted, they can easily induce teratoma formation [39]. In this paper, we aimed to display the potential of iPSC-ECs in vascular biology and regenerative medicine by analyzing the behavior of these cells both in vitro and in vivo. Furthermore, we critically reviewed the advances concerning the protocols set-up to generate and select these cells in order to overcome the most important safety issues.

## 2. Pluripotency Induction

**Figure 2:**
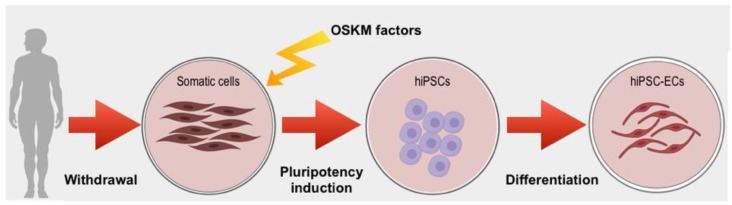
Schematic representation of human Induced Pluripotent Stem Cells-EndothelialCells (hiPSC-ECs) generation. Firstly, somatic cells are collected from the patient, then pluripotency is induced by the re-expression of four genes identified by Yamanaka et al. in 2006: OCT-3/4, Sox2, Klf4 and c-Myc (OSKM factors) which are normally inactive in somatic cells. Afterwards, induced Pluripotent Stem Cells (iPSCs) differentiation is induced towards mature Endothelial Cells (ECs).

The first method used to confer pluripotency to a somatic cell has been the nuclear transfer into an oocyte [40]. Pluripotency can be alternatively acquired by fusing a somatic cell to an ESC [41,42]. These findings indicate that both oocytes and embryonic stem cells possess factors able to confer pluripotency. Takahashi et al. identified putative pluripotency-associate genes, and among them, selected a minimum set of four genes responsible of the pluripotent state: OCT-3/4, Sox2, Klf4 and c-Myc [31] (Figure 2). These transcription factors, re-expressed into somatic cells, promote pluripotency, also affecting self-renewal and cell cycle progression [43,44,45].

To induce pluripotency the OSKM factors were introduced in somatic cells by means of viral transfection. In particular, Takahashi and Yamanaka groups, by means of Murine Leukemia Virus (MuLV) and a lentivirus delivery, were able to generate induced pluripotent cells [30]. The construct carried out by the retrovirus consisted in a single polycistronic unit under the control of an inducible promoter, while lentivirus was necessary to deliver the viral construct. This approach leads to the expression of the genetic material as soon as the inducible factor persists. Therefore, when the induction is complete, the polycistronic unit is switched off. The method described relies on the integration of the retrovirus into the genome. Genome integration is itself a limitation of this induction approach. In this context, Okita et al. demonstrated that genomic integration of reprogramming factors increases the rate of tumor formation in chimeric mice [46]. This reprogramming method is dangerous because it may cause mutations in the site of insertion and, in addition, it shows a low induction efficiency. All these aspects strongly limit the translation of the induced pluripotent cells into the clinic.

**Figure 3:**
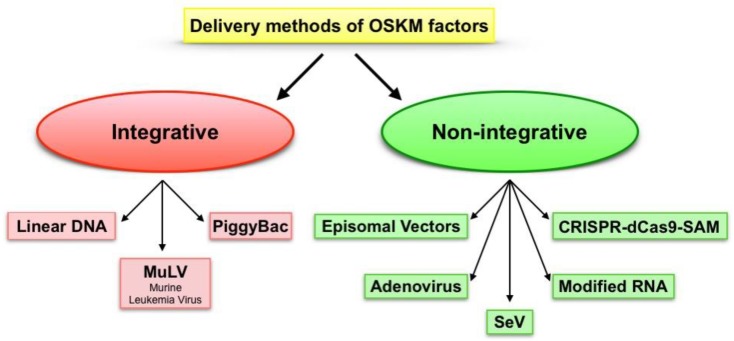
Diagram representing the different approaches to deliver reprogramming factors OSKM (Oct-3/4, Sox2, Klf4 and c-Myc) to somatic cells. The Integrative approaches: Linear DNA, MuLV (Murine Leukemia Virus), PiggyBac. The Non-Integrative approaches: Episomal Vectors, Adenovirus, SeV (Sendai Virus), Modified RNA and CRISPR-dCas9 Synergistic Activation Mediators (SAM).

Other reprogramming approaches could represent a safer alternative for clinical applications (Figure 3). In the context of integrating methods, a non-viral approach such as the transfection of linear DNA introduced by liposomes or direct electroporation can be used [47]. An intriguing approach to overcome viral delivery was developed by using PiggyBac (PB) transposon [48]. PB delivery is based on a kind of “cut and paste” mechanism by which the PB is co-transfected together with PB trasposase, causing a transgene cut from the PB vector, as well as the integration into the genomic TTAA sites. After this, the cut and paste mechanism includes a second transfection of the PB trasposase to remove the transgene from the insertion site. PB was also shown to be able to successful reprogram human somatic cells into iPSCs [49]. Despite the low efficiency, this method can be enhanced by adding butyrate to cell culture by 15- to 51-fold [50]. This approach, by involving innocuous vectors, is undoubtedly preferred to viral ones. Although PB can be considered a step forward in the development of a safe delivery method, the integration of transgene into the host DNA can cause genomic interruptions with uncontrollable downstream consequences [51].

However, non-integrative approaches are the only option suitable for the clinic in order to avoid side effects of the integrating delivery techniques.

Among others, the first choice of a non-integrative strategy is adenoviral delivery. The adenoviruses used to deliver factors are defective for replication machinery. Stadtfeld et al. indicated that such adenoviruses can reprogram somatic cells, and that no traces of integration are detected afterwards [52]. However, this approach suffers from a low infection efficiency [53]. An intriguing option in the field of non-integrative approaches was reported by Yu et al., who derived human iPS cells from fibroblasts completely free of vectors and transgene sequences by a single transfection with oriP/EBNA1 (Epstein-Barr nuclear antigen-1)-based episomal vectors [54].

Episomal vectors stem from the Epstein-Barr virus and are plasmids well suited for the introduction of reprogramming factors into human somatic cells, since they can be transfected without the need of viral packaging, and can be subsequently removed by culturing the cells without the need of drug selection [54]. The oriP/EBNA1 vectors replicate only once per cell cycle, and they can be recognized by drug selection as stable episomes in about 1% of the cells transfected [55]. The absence of drug selection causes episome loss in the ~5% of cells per cell generation due to defects in plasmid synthesis and partitioning which make the isolation of cells free of plasmids very easy [56]. Unfortunately, the efficiency of iPSC generation by episomal reprogramming remains low [57]. In 2011, Okita et al. considerably improved the efficiency (10–100 fold) of the procedure by suppressing p53 and by using non-transforming L-Myc instead of c-Myc, during the reprogramming process [58]. However, the use of the p53 short-harpin RNA (shRNA) is problematic for translational purposes, since the interference with p53 pathway may antagonize the antitumoral function of the gene [59]. In 2009, Fusaki used a Sendai virus (SeV) as a vector to generate transgene-free iPSCs in different conditions [60]. Sendai virus is a negative-strand RNA virus, differently from other RNA viruses it replicates into the cytoplasm of infected cells and does not integrate into the host genome [61]. This characteristic makes Sendai virus-based vectors the safest viral-based tool to generate iPSCs since they are considered “zero footprint” and are diluted from the infected cells with the physiological cell division [62]. To maximize reprogramming efficiency during several steps, the use of inactivated feeders, but also the use of animal-derived products, was required; however, exposure of human cells to products of animal origin increases the risks of non-human pathogen transmission and immune rejection [63]. Macarthur et al. made a step forward into a safe generation of iPSCs in 2012 [64]. These authors were able to generate, by SeV infection, transgene-free human iPSCs in feeder-free and xeno-free conditions, even though they noticed a decrease in reprogramming efficiency [64]. However, since Sendai virus vectors can reprogram with high efficiency, they were able to obtain enough colonies for further expansion.

In a recent comparison between non-integrative methods to generate iPSC, Schlaeger indicated that the SeV reprogramming approach is the most efficient and reliable, with a low workload and a complete absence of viral sequences in most lines at higher passages [65]. However, no clinical grade SeV reprogramming vectors are available. Thus, in the view of clinical applications, SeV still presents major concerns.

In this scenario the gold standard non-integrative approach is the one proposed by Warren in 2010 [66], who used modified RNA to deliver reprogramming factors. These modifications included the replacement of the 5′ cap with a synthetic one. In this protocol, RNA is complexed with cationic vehicle to facilitate cell uptake by endocytosis. Moreover, to prevent host ribonucleasic degradation and improve constructs half-life, the common cytidine and uridine bases are replaced respectively with 5 methylcitidine and pseudouridine. By these means, the authors were able to produce iPSCs [67,68]. A reprogramming method recently proposed is the one based on CRISPR-Cas9 fused to a synergistic activator mediator (SAM) [69]. This system is based on an engineered Cas9 protein (dCas9) serving as RNA-guided-DNA binding domain fused to a transcriptional activator domain (VP64). This chimeric activator complex can be directed towards promoter regions guided by specific single-guide RNAs (sgRNAs) [70]. Based on this approach Weltner et al., in 2018, generated iPSCs by targeting the promoters of OSKM factors [71].

## 3. Protocols to Induce Mature EC Phenotype

**Figure 4:**
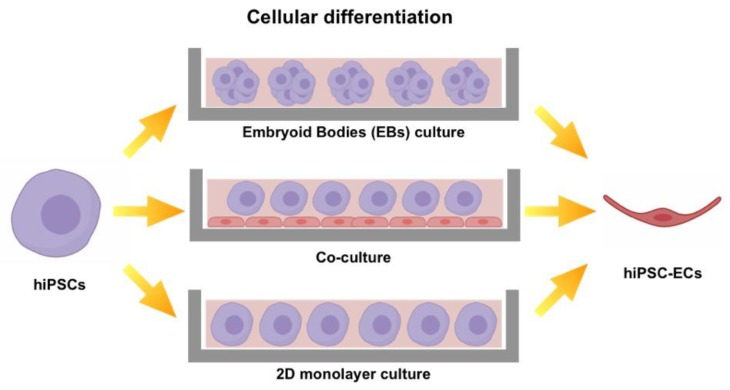
Illustration of the main strategies used to differentiate hiPSC into hiPSC-ECs. Embryoid Bodies (EBs): cultured in suspension hiPSCs tend to auto-aggregate in embryoid bodies. Co-culture: hiPSCs are co-cultured with cells able to guide their differentiation into the mature phenotype. Two-dimensional (2D) monolayer culture: hiPSCs are seeded on matrix-coated plates where they are induced to differentiate.

It is well known that for clinical use, it is mandatory to generate cells able to show a high degree of commitment. This requirement fulfills not only functional issues, but also safety ones, in order to prevent teratoma formation after implantation. Thus, a prerequisite to exploit iPSC-ECs in the clinical setting is the development of defined protocols to guide their differentiation into functional endothelial cells. The ideal induction protocol should be reproducible, easy to perform and relatively quick in order to allow yielding an adequate quantity of homogeneous cells [72]. Current induction strategies include embryonic bodies (EB) generation [73,74], differentiation on monolayers [75] and co-culture with primary cells (Figure 4).

IPSCs tend to self-assemble into three-dimensional (3D) structures (EB) when grown in suspension. From EBs, cell aggregates encompassing all three germ layers develop, and afterwards, within the positive mesodermal, EB cells tend to form vascular structures [36]. This method is affected by low efficiency (1–5%) [76] and slow production rate [77]; however, differentiation can be improved by adding proper growth factors to the culture medium [78,79]. Another approach involves a co-culture with primary cell lines able to induce iPSC-EC differentiation toward mature ECs. In detail, Choi et al. directed hiPSCs into mature ECs in the presence of OP9, a mouse bone marrow stromal cell line [80]. The authors speculated that these cells regulate iPSC induction via a paracrine signaling.

Monolayer differentiation holds a significantly higher efficiency that depends on external factors, such as medium constituents, showing a final yield that is still too low in the view of clinical applications [81]. To date, the best protocols showing the highest EC yields were developed by culturing a monolayer of hiPSCs on a matrix-coated culture plate and by treating them with different molecules or growth factors in a timed fashion in order to guide the progressive differentiation of hiPSCs toward the EC lineage [82]. In this context, GSK3 inhibitors play an important role among the set of molecules necessary to induce the differentiation of pluripotent cells into mature ECs [83]. In particular, vascular progenitors derive during human development from latero-posterior mesoderm [84]. To specify mesoderm [85,86], Wnt signaling, which is activated by GSK3 inhibition, is required [87]. In view of this, several authors have exploited GSK3 inhibitors to differentiate hiPSCs into ECs. Patsch et al. [72] exposed a monolayer of hiPSC to GSK3 inhibitor CHIR-99021 (CHIR) [88] and to mesoderm inducer bone morphogenetic protein 4 (BMP4). The combination of these two molecules led to the production of mature ECs in a relatively short time (six days) with 80% efficiency.

However, mesoderm induction is only the first step of differentiation. The second part starts upon mesodermal commitment by exposing the cells to factors that further induce the mature vascular phenotype. Gu [89] set up a protocol through which, after 4 days of treatment with VEGF and bFGF, he produced mature ECs in only 8 days. These cells, when tested, were molecularly and functionally similar to native ECs.

Paik et al. [90] added VEGF, bFGF and BMP4 to an already established protocol to produce mature ECs from hiPSCs within 12 days. Although this protocol requires more time compared to other ones reported in literature, the aim of this study was different. In fact, these cells were used to draw an RNA signature at different stage of differentiation.

The last step included in the differentiation protocol is the purification of positive cells. This step is essential to fish out a homogenous subset of cells and to ensure the safety needed for the future cell implantation and engraftment.

Cell sorting is usually performed by using magnetic beads on which surface specific antibodies are adsorbed [91]. These antibodies are directed against mature endothelial markers such as CD31 or VE-cadherin (also known as CD144).

## 4. Behavioral Differences Between iPSC and Primary ECs During In Vitro Culture

The significant vasculogenic potential of iPSC-ECs in vitro has been highlighted in several studies. Among the most noteworthy, there is the one carried out by Clayton et al., who compared three lines of endothelial cells: iPSC, induced Endothelial Cells (iECs) and cells derived from Human Coronary Artery (HCAECs) [92]. IPSC-ECs were obtained from neonatal fibroblasts through a retroviral overexpression of Oct4, SOX2, KLF4 and c-Myc and differentiated into endothelial cells. To score cell behavior concerning tubulogenesis, the authors measured cell migration and inflammatory response. Among the three cell lines (iECs, HCAECs and iPSC-ECs), the iPSC-ECs showed the best rate of vascular network formation. This result was also confirmed by cell migration assay and inflammatory response evaluation [92]. In a different study, Adams et al. compared iPSC-ECs to HUVECs by generating a human endothelium and by measuring the functional contribution of both the cell lines [93].

The results indicated that the inflammatory response, particularly the expression of cytokines and adhesion molecules as well as the number of transmigrating leukocytes, expressed by iPSC-ECs were similar to those reported for primary cells (HUVECs).

In the same study, the authors tested the electrical resistance of the cells as an indicator of the barrier function physiologically exerted by the vascular endothelium. Interestingly, iPSC–ECs displayed a lower permeability compared to HUVECs, indicating that these cells are able to create a functional endothelial barrier. This result was also corroborated by the analysis of structural protein organization that showed a better dynamic resistance of iPSC-ECs [93] when exposed to thrombin.

## 5. Exploring iPSC-ECs Features in 3D Environments

In a recent study, Campisi et al. used iPSC-ECs and primary cultures to develop an innovative 3D model of the microvascular network of the Blood Brain Barrier (BBB), able to replicate the physiologic neurovascular organization of BBB [94]. Indeed, this model displayed a selective microvasculature, with a degree of permeability lower than the one showed by the conventional in vitro models and more similar to the levels measured in rat brain.

The authors based their study on a microfluidic model comparing BBB derived from culturing human iPSC-EC alone and with a co-culture of human pericytes (PCs) and astrocytes (ACs). BBB from triple culture iPSC-ECs together with PCs and ACs showed the best performance in terms of stability and permeability.

These results indicate that iPSC-ECs co-cultured BBB functionally responds to physiological stimuli and that iPSC-ECs are a reliable model to investigate blood vessel properties in vitro [94]. Kurokawa et al. in 2017 developed a variety of methods to create a functional 3D vasculature in vitro. In particular, they compared iPSC-EC derived from CDH5-mCherry iPSCs to primary ECs [95].

After the phenotypic and functional characterization of iPSC-ECs, the authors created a microfluidic device loaded with HUVECs, Endothelial Colony Forming Cell Derived-Endothelial Cells (ECFC-ECs) and fluorescent iPSC-ECs in order to test their proliferative and vasculogenic potential.

Fluorescent iPSC-ECs displayed the physiological functions of endothelial cells when tested in standard culture condition, and a predominant venous phenotype as well as a response to shear stress when cultured into a 3D microfluidic device where they formed a perfusable vascular network. Furthermore, the cell loaded device when used for drug screening purposes showed a different behavior of the newly formed vasculature according to the different molecules used to modulate angiogenesis.

Within the microfluidic device, the authors investigated the performance of a co-culture composed of human lung fibroblasts and iPSC-ECs in comparison to the one displayed when these fibroblasts were cultured with primary ECs. More in details, they featured the characteristics of the 3D vascular network, the parameters related to the vascular barrier function, and the ability of the cells to aggregate into tube-like structures [95]. Additionally, Belair et al. described a model of engineered blood vessel developed using iPSC-EC cultured into a microfluidic device and then investigated iPSC-EC barrier function in response to a wound healing stimulus [96]. They demonstrate that human iPSC-EC reproduce functional properties of primary ECs in two in vitro platforms. After a physiological and phenotypic characterization, the iPSC-ECs and primary endothelial cells (HUVECs) were used for a comparison between Matrigel culture in standard conditions and a 3D culture in capillary-like structures embedded in fibrin. Afterwards, the authors investigated the iPSC-barrier function in response to a wound healing stimulus measured by means of impedance-based platform that records the electrical resistance as a direct indicator of cell junction damage. They generated a barrier by seeding an iPSC-EC monolayer and then assessed the expression of Zona Occludent-1 after a treatment with a low concentration of thrombin to damage the tight junctions. The authors investigated the capacity of iPSC-ECs to express cell adhesion molecules (CAMs) in response to Tumor Necrosis Factor-α stimulation, thus recapitulating EC properties necessary for cell recruitment during wound healing and inflammation. Flow cytometry analysis demonstrated that TNF-α treatment induced upregulation of Intercellular Adhesio Molecule-1 and Melanoma Cell Adhesion Molecule, which are expressed by ECs to promote attachment of immune and progenitor cells to blood vessels. The results indicate that the barrier formed with iPSC-ECs responded to a wound healing stimulus in a way that resembles mature ECs.

In the same study, the iPSC-ECs were seeded in fibrin gels into a microfluidic device and co-cultured with normal human lung fibroblasts. The co-cultured cells assembled into the three dimensional pattern developed inter-connected capillary networks and remarkably formed cord-like structures containing visibly hollow lumens [96].

## 6. Ability of iPSC-ECs to Induce In Vivo Neovascularization

Over the last decade, the behavior of iPSC-ECs in vivo has been investigated in numerous studies. Already in 2011, Li et al. revealed the ability of hiPSC-ECs to form functional blood vessels by means of tissue-engineered constructs through a Matrigel plug assay performed in immunodeficient Severe Combined Immunodeficient (SCID) mice [97]. The authors isolated endothelial cells from undifferentiated hiPSCs cultured on Matrigel-coated plates that were placed into Petri dishes with differentiation medium. Then, two weeks after the subcutaneous injection of the plugs, they harvested them and performed histological analyses revealing the presence of microvessels containing murine blood cells into their lumen [97]. In order to decrease the risk of teratoma development after implantation, Margariti et al., in 2012, generated partially induced pluripotent stem cells (PiPSC) that clearly showed the ability to differentiate into endothelial cells thanks to specific culture media and conditions. To test vessel patency and perfusion the authors used an ischemic model in SCID mice to which they injected subcutaneously a mix of PiPSC-ECs and Matrigel [98]. Fourteen days after surgery, the authors harvested the plugs and compared PiPSC-ECs with both controls (no cells) and fibroblasts reporting a significantly higher blood flow displayed by the PiPSC group. Moreover, high capillary number, stained with CD31, and a typical vascular architecture were observed in engrafted PiPSC-Ecs compared to the control group, in which the injected fibroblasts formed a random pattern. Finally, the engraftment ability was reported to be improved by using PiPSC-ECs [98]. In another very interesting study, Rufaihah et al. evaluated hiPSC-EC heterogeneity [99]. In particular, they investigated whether these cells could be characterized for each subtype. They obtained all the three principal subtypes by using different concentrations of VEGF and highlighted that arterial and venous hiPSC-ECs cytotypes were predominant. The authors injected subcutaneously into the mid lower abdominal region of SCID mice: Matrigel and bFGF (basic Fibroblast Growth Factor), heterogeneous hiPSC-ECs in Matrigel and bFGF, arterial enriched hiPSC-ECs in Matrigel and bFGF. After 14 days, Matrigel plugs were removed and immunostained with anti-CD31 Ab. Matrigel implants including hiPSC-artECs showed the ability to establish a more extensive vascular network also confirming its human origin through the positivity to human anti-CD31 immunostaining. The same work also demonstrated the onset of a widespread capillary network, especially for the arterial lineage derived from PiPSCs [99]. To further investigate and promote the use of iPSC-ECs in tissue engineering and regeneration, Clayton et al. evaluated the behavior of these cells in comparison to iECs in a mouse hind limb ischemia model. During the surgery, they made an intramuscular injection of either 1 × 10^6^ iPSC-ECs or 1 × 10^6^ iECs [92]. In particular, each treatment was divided into two injections of 25 μl (for a total volume of 50 μL of solution) on each side of the adductor muscle, nearby the area where femoral vessels had been ligated and removed. Afterwards, the authors recorded perfusion data after 0, 1, 2, 4, 7, 10 and 14 days. According to Rufaihah, at day 14 post-injection, blood perfusion was notably increased in mice receiving iPSC-ECs, although in those injected with iECs the enhancement reported at all-time points also demonstrated a high pro-angiogenic response in the short-term, specifically at day 10. Moreover, this work shows that iPSC-ECs and iECs, at day 14, were integrated with the host vasculature. Finally, at the same time-point, iPSC-ECs implanted mice did not exhibit functional increasing of capillary density in the ischemic gastrocnemius muscle with respect to mice treated with iECs [92]. Another work by Tan et al. reported that iPSCs-ECs display a longer lifetime and a higher proangiogenic function when seeded on scaffolds than when administered alone [100].

The authors injected FVB/n mice subcutaneously with: control EBM media, iPSC-ECs, iPSC-EC-seeded scaffolds and scaffolds alone. Scaffolds were composed of poly-caprolactone (PCL) and gelatin. In particular, the authors used scaffolds with a PCL:gelatin ratio of 70:30 (PG73) that supported elevated levels of iPSC-ECs growth. Indeed, by injecting iPSC-ECs seeded on PG73 scaffolds the survival of these cells increased up to 3 days.

Therefore, there was an increase of the total engraftment ability of iPSC-ECs seeded on PG73 in comparison to these cells alone. Finally, it was highlighted a higher degree of blood perfusion when the cells were included into the scaffold [100]. A further significant advance about the capacity to obtain a functional microvasculature from iPSCs has been recently made by Bezenah et al., who compared iPSC-ECs to HUVECs by co-injecting subcutaneously endothelial cells (iPSC-EC or HUVEC) and human lung fibroblasts (NHLFs) included into a fibrin matrix in CB17/SCID mice [101].

iPSC-ECs showed a consistent decrease both in vessel density and number of perfused vessels compared to HUVECs. These cells were able to form patent and perfusable vessels, showing many morphological features comparable to those expressed by HUVECs. In fact, 4, 7 or 14 days after cell injection, the authors demonstrated that iPSC-ECs/NHLF fibrin implants were able to induce vascular morphogenesis, while at days 7 and 14, the constructs exhibited increased vessel diameter and perfusion, thus confirming a high degree of integration with the host vasculature. Furthermore, vessel density increased over time in the group injected with iPSC-ECs compared to the one injected with HUVECs with a peaking value at day 7 and a decrease by day 14 [101]. Furthermore, Foster et al. used ischemic NOD-SCID mice to investigate how to control early decline viability that normally happens in iPSC-ECs after implantation [102]. The authors developed an injectable, recombinant hydrogel for cell transplantation termed SHIELD (Shear-thinning Hydrogel for Injectable Encapsulation and Long-term Delivery) able to reduce cell membrane damage during injection.

They performed intramuscular injections into the gastrocnemius muscle in the following groups of animals: PBS, SHIELD 2.5, iPSC-ECs in PBS solution and iPSC-ECs in SHIELD 2.5 They have chosen SHIELD 2.5, 2.5 wt% PNIPAM, because this formulation allows cells to proliferate over 14 days.

Animals were euthanized 14 days post-treatment and the authors demonstrated that treatment with iPSC-ECs delivered within SHIELD-2.5 resulted in significantly greater arteriole density and improved formation of large microvessels, features that usually play a prominent role in neovascularization [102]. In 2019, Ye et al. focused their attention on the exosomes derived from human iPSC-ECs (hiPSC-EC-Exo) [103]. Exosomes are vesicles containing miRNAs and are able to protect them from RNAases but they also release miRNAs which are highly involved in the regulation of angiogenesis. Indeed, it has been recently demonstrated that paracrine factors obtained from iPSCs transplantation are more effective than iPSCs themselves. Therefore, Ye et al. evaluated the role of hiPSC-EC-Exo in promoting angiogenesis in a mouse model of Peripheral Artery Disease (PAD) [103]. Immediately after the ligation of mice femoral artery, the authors injected intramuscularly either PBS or exosomes (hiPSC-EC-Exo and inhibitory-miR199b-5p-Exo) by direct injection of a total volume of 20 μL into four different sites of the ischemic hind limb. Blood perfusion was monitored at day 0, 7, 14 and 21 post hiPSC-EC-Exo treatment and an additional treatment was performed twice a week thereafter. An increased blood perfusion of ischemic limbs was shown from day 14 onwards. Then, after harvesting muscle tissue, Ye et al., by showing an increased number of CD31 positive cells, demonstrated the enhancement of neovascularization with hiPSC-EC-Exo treatment respect to the vehicle (PBS) and to inhibitory- hiPSC-EC-Exo [103]. In conclusion, to contrast several ischemic pathologies, iPSC-ECs use can bypass the shortage and the low functionality of autologous stem cells in creating a vascular network able to promote tissue regeneration. In addition, iPSC-ECs can be generated in large quantities, because they do not derive from embryos and display minimal immunogenicity. Undoubtedly, the formation of teratomas is a real risk that may result from the use of these cells; however, an approach to reduce this issue can be represented by the one proposed in the work of Margariti who created “partial-iPSC-ECs” that, during differentiation, showed reduced capacity to form teratomas [98].

Finally, the approach related to the integration of scaffolds with iPSC-ECs is very promising, especially for the capacity of the scaffolds to retain cells, implying that a lower number of cells is required to improve new vessel formation in ischemic tissues.

## 7. Conclusions

The opportunity to produce human pluripotent cells (hiPSCs) from somatic cells is one of the most exciting breakthroughs of this scientific era. HiPSCs are useful to develop patient-specific drug screening and validation methodologies as well as to model human diseases, thus allowing to shape an individualized cell therapy. In view of this, these cells clearly represent the launching pad for an efficient personalized medicine.

First-in-Human (FIH) test have been performed in 2014 to treat age-related macular degeneration [104]. Mandai et al. transplanted an autologous iPSC-derived retinal pigment epithelium (RPE) cell sheet [104]. The study involved two patients. Patient 1 did not show any serious adverse event 25 months after implantation, meaning that the procedure did not trigger the host immune response, nor did it trigger tumor formation [104]. Patient 2 did not complete the procedure due to the detection of deletions in chromosome X patient derived iPSCs. Although tumorigenicity has never been reported in association to these deletions, the team decided to exclude patient 2 from the trial. In any case, the same cells implanted in mice did not develop teratomas. It is evident that iPSCs carry on intrinsic critical points that should be carefully analyzed. Reprogramming itself can lead to genetic and epigenetic dysregulation. As previously mentioned, some reprogramming factors are potent oncogenes [105], and it has been widely reported that the reactivation of these genes is able to cause teratoma formation. Another significant issue that should be addressed is the removal from transplanted iPSC of those cells that are not completely differentiated [106], thus implying a careful selection and an accurate screening of the cells. In this respect, in vivo teratoma assay is still an expensive and time-consuming procedure. A molecular approach based on Quantitative Reverse-Transcription Polymerase Chain Reaction (qRT-PCR) could be useful holding the sensitivity needed to detect undifferentiated cells [107]. More in detail, this assay relies on the detection of Lin28, a pluripotency-associated gene, used to recognize undifferentiated cells.

In conclusion, in this work, we reviewed the potential hold by hIPSC-ECs in providing an efficient alternative to primary cells for regenerative medicine applications. The use of IPSC-EC as vascular forming cells is encouraged by several studies involving the comparison with well-established cell lines both in vitro and in vivo. Due to their abilities, it is not difficult to imagine a widespread use of iPSC-EC as the preferential source of endothelial cells in tissue engineering. However, the main issue concerning the safety of their use in the clinic persists. In view of this, to fully exploit hiPSC-ECs potential, it is mandatory to set up reliable methods for their production in order to fulfill clinical grade requirements. The current protocols to induce pluripotency, guide the cells towards a mature phenotype and effectively select the resulting cells seem to be ready to go from bench to clinical trials [108].
